# Supplementary figures and images for: High-quality whole-genome sequence analysis of Lactobacillus paragasseri UBLG-36 reveals oxalate-degrading potential of the strain
Source: PLoS One. 2021 Nov 19;16(11):e0260116. doi: 10.1371/journal.pone.0260116 (PMC8604369; doi:10.1371/journal.pone.0260116)

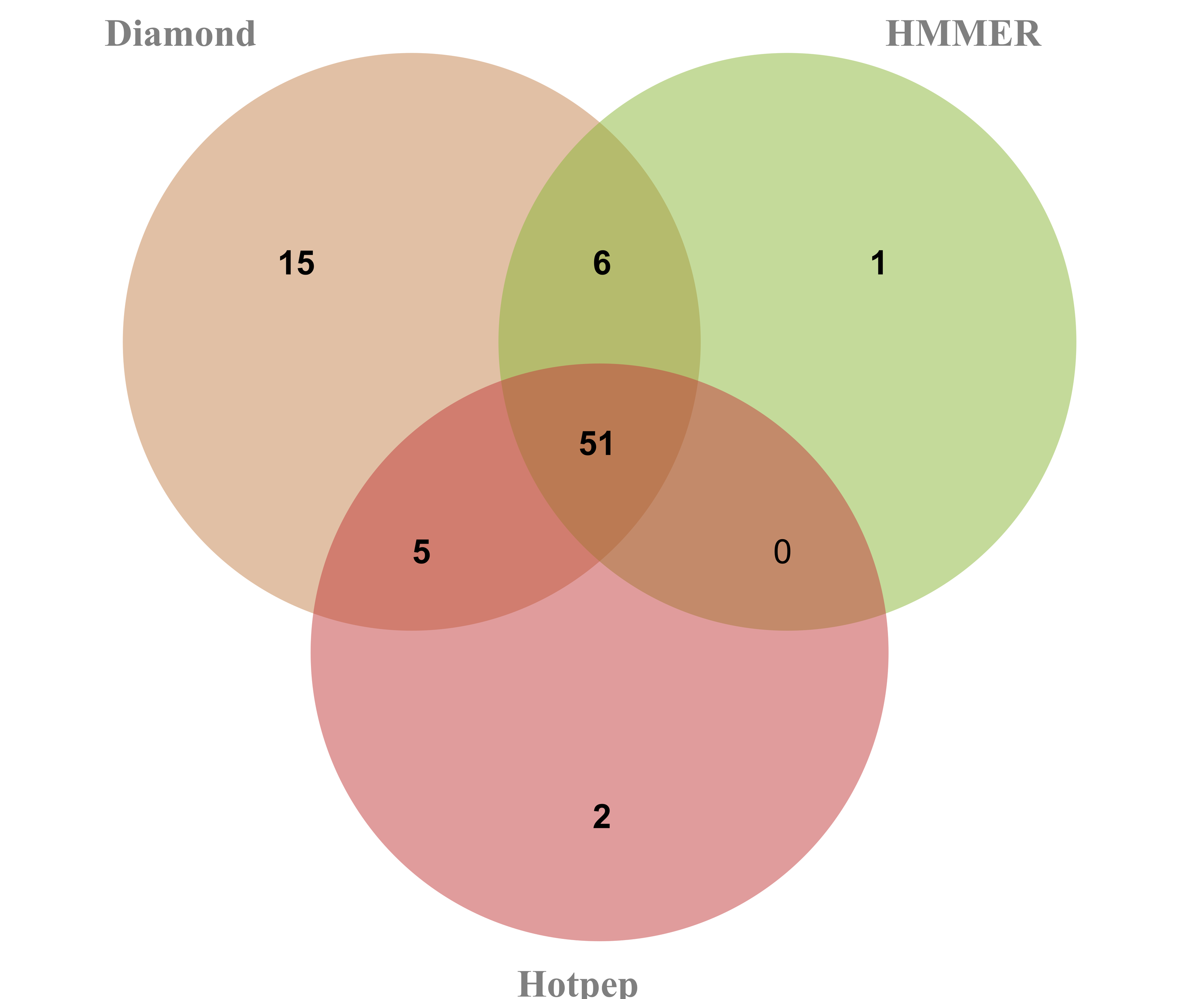

Supplement: S1 Fig — (TIF) [file pone.0260116.s001.tif]
