# Supplementary material for: High-quality whole-genome sequence analysis of Lactobacillus paragasseri UBLG-36 reveals oxalate-degrading potential of the strain
Source: PLoS One. 2021 Nov 19;16(11):e0260116. doi: 10.1371/journal.pone.0260116 (PMC8604369; doi:10.1371/journal.pone.0260116)
Supplement: Attachment — Submitted filename: Response to reviewers.docx [file pone.0260116.s008.docx]

**Response to reviewers**

We would like to thank the reviewers for their thorough reading of the manuscript and the thoughtful comments and constructive suggestions. Acting on the revisions prompted by the reviewers’ helped in improving the quality of the manuscript. Changes in the revised manuscript has been done with track change option in MS Word.

**Reviewer 1:**

**Comment 1:** When performing the initial phylogenomic analysis the complete genomes of 4 *L. paragasseri* and 4 *L. gasseri* strains were used. There are currently 8 genomes for *L. gasseri* strains on NCBI, why not use them all?

**Response:** We could not use the new L. gasseri strains, namely, HL20, HL70, and HL75, as they were not available at the time, we performed the analysis. Further, ANI blast analysis on strains 4M13 and MGYG-HGUT-02387 indicated identical (duplicate genomes). Therefore, we only included MGYG-HGUT-02387 in our analysis. However, we thank the reviewer for his suggestion, and in our revised manuscript, we have included these new strains and performed a new set of phylogenomic analyses (see section “Phylogenomic analysis” under “Results and Discussion”).

**Comment 2**. In figure3, there are too many colors for the legends and it is making it a bit difficult to differentiate between the positive negative controls and the different time points. Maybe use more labels on the graph to make the figure easier to comprehend.

**Response:** As per the reviewer’s suggestion, we have modified the figure to make it more legible and comprehensible (See Fig3).

**Reviewer 2:**

**Comment 1:** Should there be any concerns that these antibiotic resistance genes are present (in accordance with EFSA regulations)? Any flanking regions that suggest possible transfer of resistance genes?

**Response:** We thank the reviewer for raising this concern, as it prompted us to investigate these genes more closely. We realized that our initial assessment of antimicrobial-resistant genes (AMR) using PATRIC was wrong because the output of PATRIC is based only on the homology of the query sequence with genes of those organisms that are in the PATRIC AMR gene database (which does not include any *Lactobacillus* or probiotic species). Moreover, we realized that the PATRIC database is not the best tool to identify putative AMR genes as PATRIC only indicates the possible AMR mechanisms and does not provide the genes that can putatively confer AMR. Therefore, we repeated the analysis by submitting the draft genome of UBLG-36 to the ResFinder tool. No, AMR genes were identified in the genome of UBLG-36. To confirm this data, we aligned the annotated sequence of UBLG-36 against the protein sequences of AMR genes in the Comprehensive Antibiotic Resistance Database (CARD). Similar to ResFinder, no resistance phenotype or gene was observed in UBLG-36 when searched in the CARD database. We have, therefore, updated this information in both the “Materials and Method” and the “Result and Discussion” sections of the revised manuscript and sincerely apologize for this mistake.

**Comment 2:** Why is carbohydrate metabolism an important trait and what might this imply towards the environmental niche whereby UBLG-36 is expected to survive? Does the CAZyme analysis imply preference for particular substrates?

**Response:** While we were not emphasizing any particular substrate in our analysis, in our revised manuscript, we have included some brief points as to how the presence of these enzymes might be beneficial to UBLG-36 (see section “Carbohydrate-active enzymes (CAZymes)”). For example, Carbohydrate metabolism provides the main source of metabolic energy in LAB. It plays an important role in the survival and fitness of *Lactobacillus* in their ecological niche by contributing to cellular processes such as energy production and stress response. We believe that the presence of these enzymes will aid UBLG-36 in its survival, competitiveness, and persistence within the host.

**Comment 3:** What are the implications of the discovery of these bacteriocin producing clusters? Are these expected from *L. gasseri* / *L. paragasseri*?

**Response:** As mentioned in the “result and discussion” of the manuscript under the “Secondary Metabolites”, bacteriocins are essential antimicrobial peptides produced by LAB. Of particular interest amongst all classes of bacteriocins are the class V circular bacteriocins such as gassericin reported in our strain, *L*. *paragasseri* UBLG-36. As mentioned in the revised manuscript (line 233, “Secondary metabolites”), bacteriocins like gassericin are simple representatives of ubiquitous circular peptides with diverse physiological activities (Craik et al. 2006 *Biopolymers* 84:250–266). They were first identified in *L. gasseri* LA39 and have been shown to be active against several foodborne pathogenic bacteria (Kawai et al. 2001 *Food Microbiol* 18:407–415). As *L. paragasseri* strains are a sister taxon of *L. gasseri*, it is expected that *L. paragasseri* strains will also have bacteriocin clusters in their genome as described previously (see reference 9 in the revised manuscript).

**Comment 4:** While the title of the manuscript refers to the oxalate degrading potential of UBLG-36, there is an overall lack of emphasis in the text that describes the importance of oxalate degradation and why this is the focus of the manuscript. Perhaps, a few sentences are needed to highlight the importance of this trait and how it is valuable in probiotics and human health. In addition, it will be worth mentioning the general capability of probiotic LAB species in oxalate degradation and how this trait differs between species/strains.

**Response:** We agree with the reviewer’s comment that the manuscript lacks an emphasis on the importance of oxalate degradation, both in general and in the context of *L. paragasseri* UBLG-36. Therefore, in the revised manuscript, we have included several points under “Introduction” and the “Results and Discussion” sections highlighting the relevance of discovering oxalate degrading genes in UBLG-36 and the necessity for oxalate degradation.

**Comment 5:** Besides, additional pangenomics analyses can be carried out between the 8 selected genomes (or more if publicly available), especially in the context of the *oxc* and *frc* genes that are of main interest in this manuscript. This should provide insights as to how frequent these oxalate degradative genes are present in *L. gasseri / L. paragasseri* species (highly conserved?) If these are already known from previous publications, it would be worth citing and discussing.

**Response:** Although we understand that pangenomics results will be insightful, we are currently using the pangenomics data of UBLG-36 to perform several transcriptional and functional analyses on the strain in our laboratory. Therefore, we apologize for being unable to provide a comparative pangenomic analysis between UBLG-36 and other type strains, in this manuscript, at this point. However, in the “Introduction” to our revised manuscript, we have discussed few key points from a recently published pangenomic study by Zhou et al. (2020) compared the genetic diversity of 13 strains of *L. gasseri* and 79 strains of *L. paragasseri* (Line 76, see reference 9 in the revised manuscript). Further, we thank the reviewer for the suggestion and have performed analyses to determine the evolutionary conservation of *oxc* and *frc* between *L. gassei* and *L. paragasseri* species using complete genomes present in the NCBI database. We have included this new detail in the “Materials and Methods” and have discussed the results in the revised manuscript (See line 125 and 308, and Fig 4 in the revised manuscript)

**Comment 6:** Fig3: Statistical analysis required to show significant differences between UBLG-36 with the controls and between the two different concentrations at each time point.

**Response:** As per the reviewer’s suggestion, we have performed the statistical analysis and have updated the information under “Results and Discussion” in the revised manuscript (Line 294 and Fig 3)

**Comment 7:** It would be insightful to discuss if the percentage of oxalate degradation reported here are consistent with other probiotic *L. gasseri*/*L. paragasseri*/LAB species that were previously reported, as oxalate degradation activity is highly variable (Turroni et al. 2007, Azcarate-Peril et al. 2008). With some strains reported to have 100% oxalate degradative activity, where does UBLG-36 stand?

**Response:** We agree with the reviewer that some discussion on the presence of oxalate degrading genes and the results of oxalate degradation by UBLG-36 are necessary. Hence, in the revised manuscript, we have added a concise paragraph discussing the consistency of our results with other LAB strains (Line 325). We agree that the degree of oxalate degradation by LAB is highly variable and believe that it is most likely due to species- and strain-specific differences. Further, in their study, Turroni et al. (2007) showed that oxalate degradation as much as 40% was an effective trait in *L. gasseri,* although some strains showed 100% degradation. However, their study measured oxalate degradation over five days by inoculating the bacteria in 5 mM oxalate. Few significant differences in our study (apart from the strains used) is that UBLG-36 was subjected to higher concentrations of oxalate, and we have measured oxalate degradation for a longer duration than that reported by Turroni et al. Therefore, the notable differences in the levels of degradation between UBLG-36 and other *L. gasseri* or LAB species may not only be influenced by the type of strain or species but also the experimental setup. Also, to the best of our knowledge, this is the first report on the ability of an *L. paragasseri* strain in degrading oxalate. Therefore, based on the current results, we believe that UBLG-36 is an oxalate degrader with high potential and can show promising results when characterized further.

**Comment 8:** It appears that there are opposing trends between DSM 20079 and UBLG-36 with respect to different oxalate concentrations at all three timepoints. For DSM 20079, the percentage of oxalate degradation appears to be higher at 20mM compared to 10mM while for UBLG-36, the percentage of oxalate degradation appears to be lower at 20mM compared to 10mM. Was degradation at 20mM statistically different compared to 10mM for each strain? If yes, is there a proposed hypothesis as to why this difference in dose-dependent effects were observed?

**Response:** The opposing trends in oxalate degradation between DSM 20079 and UBLG-36 maybe due to species-wise differences in catabolizing oxalate. Such variability has been previously reported (Turroni et al. 2007, Azcarate-Peril et al. 2008). Further, as far as dose-dependent effect is concerned, the differences in the physiological state and adaptation of the cells to the oxalate medium may also be contributing to the observed effect. We want to add that DSM 20079 was only used as a positive control for the experiment (because it harbors *oxc* and *frc*), and our analysis did not compare this strain’s effectiveness against UBLG-36. However, as per the reviewer’s suggestion, in the revised manuscript, we have shown the statistical analysis (Line 294 and Fig 3) where we have statistically analyzed the difference in oxalate degradation between UBLG-36 and DSM 20079 (positive control) on all three-time points. We have also analyzed the oxalate degradation by UBLG-36 at 10 mM and 20 mM MRS-Ox at different time points. There was no significant difference in oxalate degradation by UBLG-36 on day 1 between 10 mM and 20 mM MRS-Ox. However, when incubated in 20 mM MRS-Ox, the average degradation significantly reduced on days 5 and 10 compared to that observed in 10 mM MRS-Ox on the same days. Further, the percentage oxalate degradation in 10 mM and 20 mM MRS-Ox by UBLG-36 was significantly higher than the positive control, *L. acidophillus* DSM 20079, at all three-time points.

**Comment 9:** Briefly expand on what are some of the important assays/phenotyping that are needed to establish a strain as a probiotic.

**Response:** As per the reviewer’s suggestion, we have briefly mentioned in our discussion few important functional assays that are necessary to classify a strain (or, in this case, UBLG-36) as a probiotic (Line 336)
