# Supplementary material for: High-quality whole-genome sequence analysis of Lactobacillus paragasseri UBLG-36 reveals oxalate-degrading potential of the strain
Source: PLoS One. 2021 Nov 19;16(11):e0260116. doi: 10.1371/journal.pone.0260116 (PMC8604369; doi:10.1371/journal.pone.0260116)
Supplement: Attachment — Submitted filename: Response to reviewers.docx [file pone.0260116.s009.docx]

**Response to reviewers**

We would again like to thank the reviewers for their thorough review of the manuscript and their constructive suggestions. Although the changes were minor, acting on the revisions prompted by the reviewers has immensely improved the manuscript. Changes in the revised manuscript have been done with the track change option in MS Word.

**Reviewer 2:**

**Comment 1:** Line 141-It is advisable to state the factors used for the two-way ANOVA. Was a two-way or one-way ANOVA used for comparison in Fig 3a? It is advisable to include the negative control in the statistical analysis.

**Response:** We thank the reviewer for suggesting these changes. One-way ANOVA was used in figure 3a, and two-way ANOVA was used in figure 3b. We have stated these factors in the revised manuscript (line 140 under “Materials and methods”). Further, we have included the negative control in the statistical test, and the new figure 3 reflects these changes.

**Comment 2**. Line 305-Include the fact that no oxalate degradation was observed after indicating that *L. casei ATCC 334* served as a negative control.

**Response:** As per the reviewer’s suggestion, we have included a line that *L. casei* 334 was used as the negative control and showed no oxalate degradation. (See line 306).
